# Tribute to Dr. Caldwell

**Published:** 1853-08

**Authors:** 


### THE WESTERN JOURNAL.

THIRD SERIES.—VOL. XII.—NO. II.

LOUISVILLE, AUGUST, 1853.

### TRIBUTE TO DR. CALDWELL.

The death of Dr. Caldwell is announced in another part of this Journal. The following proceedings growing out of that event will be read with interest:

At a meeting of the physicians of Louisville held on the 12th July, 1853. Dr. J. W. Knight was called to the chair, and Dr. B. I. Raphael appointed secretary.

On motion of Dr. J. J. Mathews, a committee of three was appointed to draft resolutions expressive of our feelings in reference to the death of our venerable friend and fellow-citizen, Dr. Chas. Caldwell.

The following gentlemen were appointed by the chair on said committee: Dr. J. J. Mathews, Dr. T. W. Colescott, and Dr. H. Chenoweth.

The committee having retired, returned in a short time and presented the following report, which was unanimously adopted:

It having pleased an all-wise Providence to call from the scene of his earthly labors, the venerable Dr. Chas. Caldwell, the most widely known and the most eminent member of the medical profession in the Union, endeared to us in the three fold relation of neighbor, friend, and teacher, by the blended dignity and courtliness of his manner, the purity of his life, his profound and varied learning, and his indefatigable labors for the space of two generations in the cause of science and humanity; therefore—

*Resolved.* That we have received with profound regret the intelligence of the death of Dr. Caldwell.

*Resolved,* That, in the death of Dr. Caldwell, the American Medical profession has lost one who, while living, most illustrated its annals by his talents and adorned it by his virtues; medical science one of its oldest and most ardent votaries and medical philosophy the most distinguished and successful interpreter that our country has yet produced.

*Resolved,* That with a profound sense of tne vast services rendered by Dr. Caldwell to the science of medicine, and the great interests of society, we will not cease to cherish and to guard his memory with affectionate respect and admiration.

*Resolved,* That we tender to the bereaved family of the deceased our warmest sympathy, and that the secretary be directed to transmit to them a copy of these resolutions.

On motion it was—

*Resolved,* That Drs. Raphael, Hardin, and Ronald be constituted a committee to select a suitable person to deliver a eulogy on the deceased.

It was further—

*Resolved,* That these proceedings be published in the daily papers of the city, in the Western Journal of Medicine and Surgery, and the Transylvania Journal of Medicine.

On motion, the meeting adjourned *sine die.*

J. W. Knight, Chairman.

B. I. Raphael, Secretary.

